# Arthroscopic Iliopsoas Lengthening Is a Safe and Effective Treatment for Anterior Iliopsoas Impingement After Total Hip Arthroplasty

**DOI:** 10.1016/j.asmr.2025.101262

**Published:** 2025-09-17

**Authors:** James Pate, Austin Hughes, Dillon Morrow, Tyler M. Goodwin, Andrew Wilson, Brandon Cincere

**Affiliations:** a Department of Orthopedic Surgery, University of Tennessee at Chattanooga, Chattanooga, Tennessee, U.S.A.; b Department of Orthopedic Surgery, Florida International University/Baptist Health South Florida, Miami, Florida, U.S.A.

## Abstract

**Purpose:**

To assess the demographic characteristics, preoperative variables, and postoperative outcomes of patients who underwent arthroscopic iliopsoas lengthening for anterior iliopsoas impingement (AII) after total hip arthroplasty (THA).

**Methods:**

A retrospective, single-surgeon case series was conducted to identify patients with AII after THA who underwent arthroscopic iliopsoas lengthening between 2017 and 2024. A minimum 1-year follow-up after arthroscopic procedures was required for patients to be included in this study. All arthroscopic procedures were completed by a single orthopaedic sports fellowship-trained surgeon. The primary outcomes were the incidence of THA revision, incidence of reoperations and secondary surgical procedures, and changes in pain scores.

**Results:**

Of 15 hips, 12 (80%) were reported to be pain free at most recent follow-up (median follow-up, 3.8 years [range, 1.3-6.8 years]). There were no THA revisions performed after arthroscopy, no reoperations or secondary surgical procedures, and no infections requiring surgical intervention. The median pain score decreased from 8 preoperatively (interquartile range, 6.5-10; range, 3-10) to 0 postoperatively (interquartile range, 0-1; range, 0-5) (*P* < .001). For the index THA, a posterior approach was used in 7 patients (47%) whereas an anterior approach was used in 8 (53%).

**Conclusions:**

Arthroscopic iliopsoas lengthening is a safe and effective treatment for AII after THA. The procedure had minimal complications, provided considerable pain relief in 80% of patients, and helped avoid major revision arthroplasty surgery in 100% of cases.

**Level of Evidence:**

Level IV, retrospective therapeutic case series.

A previously under-recognized sequela of total hip arthroplasty (THA) is the development of anterior iliopsoas impingement (AII) and tendinitis.^1^ One study suggested that AII may be the cause of persistent pain after THA in 4.3% of cases.^2^ A recent retrospective insurance database study using Current Procedural Terminology (CPT) and *International Classification of Diseases, Tenth Revision* codes, comprising 314 patients, examined iliopsoas pathology among patients undergoing arthroscopy after THA.^3^ *International Classification of Diseases, Tenth Revision* diagnosis codes for iliopsoas tendinitis were found in 105 of 314 patients (33.4%). In addition, a more comprehensive systematic review suggested similar data, with an iliopsoas tendinitis rate of 35.8%.^4^ Several sources of impingement have been proposed, including prominent or malpositioned acetabular components, aberrant placement of screws in the acetabular cup, a proud cement mantle, heterotopic ossification, or the use of a cage construct.^5^ The most common site of AII is thought to be at the anterior rim of the acetabulum, causing inflammation and tendinopathy of the adjacent iliopsoas tendon.^6^ Other causes of irritation of the iliopsoas after THA have been described, such as increased offset or excessive lengthening of the leg.^7^

Conservative management using nonsteroidal anti-inflammatory drugs (NSAIDs), physical therapy, and recurrent peritendinous injections of corticosteroids has been proposed and should be considered prior to any surgical intervention, although it has not shown consistent prolonged success.^8^ Iliopsoas lengthening, revision of the acetabular component, and trimming of metal phalanges are all surgical treatment strategies that have been described with some success.^2^^,^^9^^,^^10^ Dora et al.^5^ showed pain relief after iliopsoas lengthening or acetabular component revision in 81.8% of patients in whom conservative management had previously failed.^2^ The acetabular revision group showed similar functional scores at final outcome assessment but had a significantly higher complication rate compared with the iliopsoas lengthening group.

Iliopsoas lengthening has historically been performed through an open approach, with more recent literature describing the safety and efficacy of endoscopic and arthroscopic iliopsoas lengthening.^11^ Endoscopic lengthening involves tenotomy at the level of the lesser trochanter,^12^ whereas arthroscopic lengthening consists of a trans-capsular release at the level of the psoas notch.^13^ A recent systematic review evaluated the outcomes after endoscopic and arthroscopic treatment.^14^ Among the 7 studies evaluating endoscopic technique, the average rate of favorable outcomes was 81.3% (range, 62%-97.2%). Among the 7 studies that used a trans-capsular arthroscopic technique, the average favorable outcome rate was 89.4% (range, 81%-100%).

The purpose of this study was to assess the demographic characteristics, preoperative variables, and postoperative outcomes of patients who underwent arthroscopic iliopsoas lengthening for AII after THA. We hypothesized the iliopsoas lengthening would provide a predictable decrease in visual analog scale pain scores in patients with AII.

## Methods

All arthroscopic procedures were completed by a single orthopaedic sports fellowship-trained surgeon (B.C.) at a single institution between January 2017 and January 2024. Inclusion criteria were age 18 years or older with a history of THA and diagnosis of iliopsoas impingement. Common procedural codes were used to include patients, consisting of CPT code 27005 (open tenotomy of the hip flexors), CPT code 1005648 (hip arthroscopy), CPT code 29863 (hip arthroscopy with synovectomy), and CPT code 29861 (hip arthroscopy with removal of loose body or foreign body). A minimum 1-year follow-up after arthroscopic surgery was required for patients to be included. Patients younger than 18 years, those without a history of THA, and those with less than 1-year follow-up were excluded.

**Fig 1:**
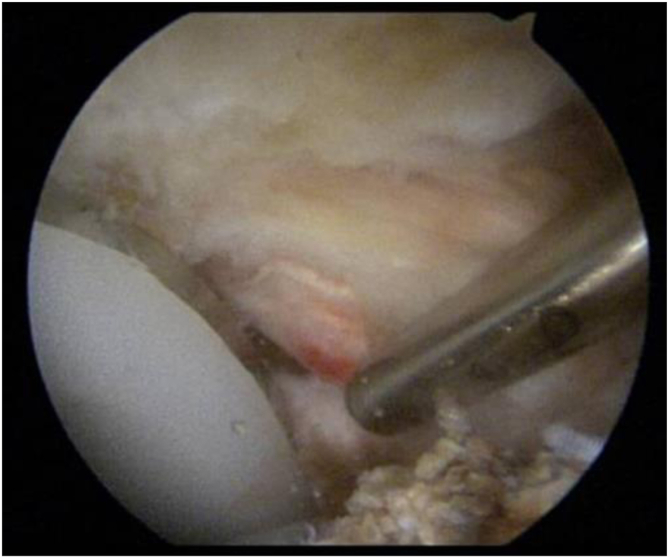
Arthroscopic view of a right hip from the standard anterolateral viewing portal showing heterotopic bone formation at the anteromedial edge of the cup with inflammation and tendinopathy of the adjacent iliopsoas tendon.

**Fig 2:**
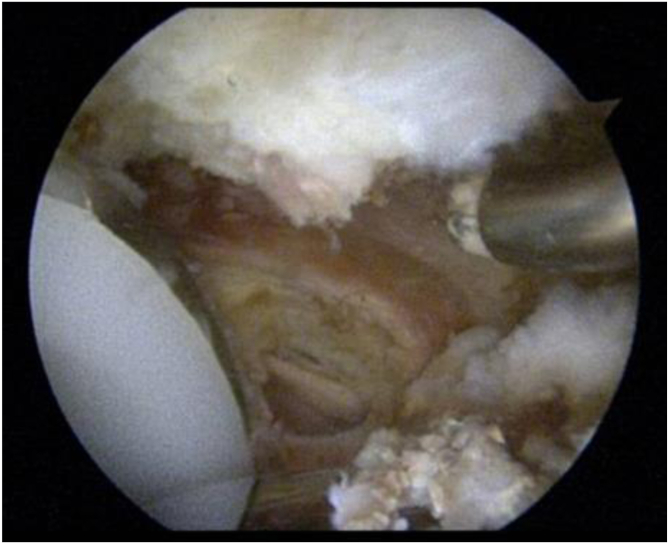
Arthroscopic view of a right hip from the standard anterolateral viewing portal after arthroscopic release of the iliopsoas tendon and debridement of the heterotopic bone adjacent to the anteromedial edge of the cup.

After institutional review board approval (University of Tennessee College of Medicine Institutional Review Board No. 2223831) was obtained, a retrospective chart review was performed to identify consecutive patients with painful THA who subsequently underwent hip arthroscopy. The indications for surgery used for the case series were symptoms of AII after previous THA. Iliopsoas lengthening was performed arthroscopically at the level of iliopsoas impingement at the anteromedial rim of the acetabulum. The iliopsoas was lengthened from the central compartment through the tendon only, leaving the muscle intact, and any heterotopic bone was shaved down or excised, as shown in Figures 1 and 2.

Patient characteristics, surgery details, follow-up data, and complication rates were collected and are reported in Table 1, Table 2, Table 3 to Table 1, Table 2, Table 3. The primary outcomes were the incidence of THA revision, incidence of reoperations and required secondary surgical procedures, and changes in the visual analog scale pain measurement (0-10). Statistical analyses were performed with R, version 4.4.1 (The R Foundation). Comparison of preoperative and postoperative pain scores was conducted with the Wilcoxon signed rank test.

**Table 1:** Demographic Characteristics of Patients Who Underwent Arthroscopic Iliopsoas Lengthening After Total Hip Arthroplasty

	Data
Total patients	15
Total hips	15
Age, mean (SD), yr	58 (11)
Sex, n (%)	
Female	7 (47)
Male	8 (53)
Body mass index, mean (SD)	28.7 (5.5)
Cigarette smoker, n (%)	8 (53)
Diabetes, n (%)	2 (13)
Neuropathy, n (%)	4 (27)
Osteopenia, n (%)	0 (0)
Osteoporosis, n (%)	0 (0)
Peripheral vascular disease, n (%)	0 (0)

SD, standard deviation.

**Table 2:** Preoperative and Postoperative Details of Patients Who Underwent Arthroscopic Iliopsoas Lengthening After THA

	Data
Total patients	15
Total hips	15
Duration of symptoms, median (range), yr	1.5 (0.3-8.5)
Time between THA and arthroscopy, median (range), yr	2.4 (0.5-8.5)
Approach and cementation at index THA, n (%)	
Posterior approach	7 (47)
Anterior approach	8 (53)
Cemented	1 (7)
Non-cemented	14 (93)
No postoperative pain	12 (80)
Reoperation, n (%)	
Total hip arthroplasty revision	0 (0.0)
Infection	0 (0.0)

THA, total hip arthroplasty.

**Table 3:** Changes in Visual Analog Scale Pain Scores After Arthroscopic Iliopsoas Lengthening

	Preoperative	Postoperative	*P* Value
Pain score, median (interquartile range)	8 (6.5-10)	0 (0-1)	<.001

## Results

During the study period, hip arthroscopy was performed on 15 hips of 15 consecutive individuals with symptomatic THA by 1 fellowship-trained sports medicine orthopaedic surgeon. The overall follow-up rate was 100%, with no patients lost to follow-up. The median length of clinical follow-up was 3.8 years (range, 1.3-6.8 years). The median time between the most recent arthroplasty procedure and arthroscopy was 2.4 years (range, 6 months to 8.5 years). The median duration of symptoms prior to intervention was 1.5 years (range, 4 months to 8.5 years). The average age was 58 years (standard deviation, 11 years), the average body mass index was 28.7 (standard deviation, 5.5), and 8 patients were male (53%). There were 8 were cigarette smokers (53%), 2 patients had diabetes (13%), 4 patients had a diagnosis of neuropathy (27%), and 2 patients were undergoing THA revision (13%). Demographic variables are shown in Table 1. The approach used for previous THA consisted of a posterior approach in 7 patients (47%) and an anterior approach in 8 (53%). One patient (7%) had a cemented THA, whereas 14 patients (93%) had non-cemented THAs. When initially discharged, 12 patients (80%) were allowed weight-bearing as tolerated (WBAT) after arthroscopy, 1 patient was allowed WBAT 2 weeks after surgery, and 2 patients were allowed WBAT 4 weeks after surgery. During follow-up, there were no THA revisions, reoperations and secondary surgical procedures, or infections requiring surgical intervention. Duration of symptoms, approach used, and postoperative outcomes are shown in Table 2.

The median pain score decreased from 8 preoperatively (interquartile range, 6.5-10; range, 3-10) to 0 postoperatively (interquartile range, 0-1; range, 0-5) (*P* < .001). These results are shown in Table 3. Of 15 patients, 12 (80%) reported having no pain at the most recent follow-up. One patient, a male smoker with persistent postoperative pain, had a prior THA revision with 2-stage infection treatment 2 years before the arthroscopy procedure. Seven months after arthroscopy, the patient was experiencing persistent pain (5 of 10) with minimal improvements in symptoms, he had no implant or misalignment issues, and bone scan findings were negative for infection or loosening. Nonoperative treatment was pursued, and there were no additional surgical procedures at 4.5 years. A second patient, a neuropathic male patient with a prior THA revision and persistent postoperative pain (4 of 10), had ischiogluteal bursitis, was treated with platelet-rich plasma injections, and had no subsequent reoperations 2 years after arthroscopy. He reported satisfaction with the postoperative improvements in pain and function. A third patient, a female smoker with diabetes, was still experiencing pain (3 of 10) 2 years after arthroscopy and had no additional surgical procedures. She reported satisfaction with the postoperative improvements in pain, function, and her ability to be more active. Table 1 shows descriptive characteristics, outcome values, and complications as mentioned earlier.

## Discussion

This study shows the clinical utility of arthroscopic iliopsoas lengthening in the treatment of AII after THA, with 12 of 15 of patients having no pain at their most recent follow-up. There was also a considerable reduction in pain scores postoperatively compared with preoperatively, with the median pain score improving from 8 preoperatively to 0 postoperatively (*P* < .001). This procedure was tolerated very well with low rates of complications. Currently, there is no evidence in the literature referencing the chance of recurrence of symptoms of iliopsoas impingement after 1 year of being asymptomatic. With a minimum follow-up period of 1 year and median follow-up period of 3.8 years, ample time was given to assess recurrent symptoms. There were no subsequent THA revisions, reoperations and secondary surgical procedures, or infections requiring surgical intervention.

AII after THA is an uncommon problem that can be difficult to diagnose and treat. Twelve millimeters of overhang of the acetabular cup is a sensitive and predictive parameter for the diagnosis of AII.^15^ As mentioned earlier, conservative treatment with NSAIDs, physical therapy, and peritendinous iliopsoas corticosteroid injections has limited success at achieving prolonged symptom relief.^8^ Iliopsoas impingement has been shown to be the most common indication for hip arthroscopy after THA.^4^ The French Arthroscopy Society performed a prospective, multicenter endoscopic and arthroscopic iliopsoas lengthening study in patients with AII after THA.^11^ Of the 64 patients who underwent arthroscopic or endoscopic iliopsoas lengthening, 92% reported alleviation of pain, and 87% of patients were satisfied. These data add to the limited body of literature supporting the efficacy and safety of arthroscopic iliopsoas lengthening for AII. Despite recent literature showing successful results with minimal complication profiles, this procedure has been slow to be adopted by the orthopaedic community. As a result, patients often experience delayed care or undergo unnecessary revision surgery. Acetabular cup revision has been shown to have higher complication rates, often without resolution of symptoms, as compared with iliopsoas lengthening.^5^ The results of this study demonstrate this phenomenon, with a median time between THA and iliopsoas lengthening of 2.4 years (range, 6 months to 8.5 years).

Buller et al.^16^ reported on several factors indicated in predisposing patients to AII, including female sex, small native femoral head diameter, increased acetabular component–to–femoral head diameter ratio, and any measurable acetabular component overhang. Their study assessed 559 hips after a direct anterior approach exclusively, with an average follow-up period of 56 months. The authors reported the incidence of AII after the direct anterior approach to be approximately 6%. In comparison, prior studies reported the incidence after the posterior approach to be approximately 4%.^5^^,^^9^ Although the sample size is small, our cohort results show a similar incidence of AII with both anterior (n = 8) and posterior (n = 7) approaches.

**Fig 3:**
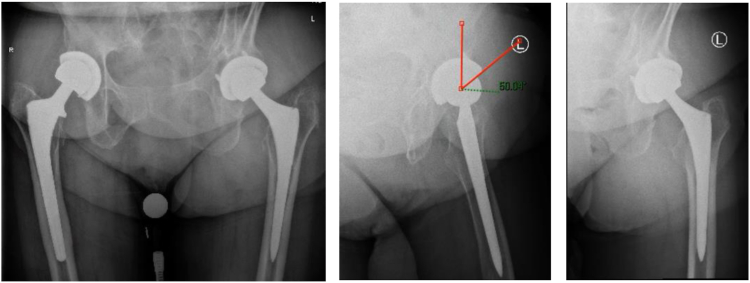
Low-set anteroposterior, false-profile, and 45° frog-leg lateral views of a left hip showing significant anterior protrusion of the acetabular cup. No obvious heterotopic ossification or hardware complications are noted. The anterior center-edge angle on the false-profile view is noted to be 50° as denoted by red lines (dotted green line denoting angle between red lines), with 15 mm of acetabular cup protrusion. (L, left; R, right.)

Regardless of the approach used, we believe that it is important to address the pathology at the site of impingement. This is often at the anteromedial rim of the cup adjacent to the iliopsoas, and it is important to address any bony impingement in the form of heterotopic ossification, as shown in Figures 1 and 2. The literature often reports the use of cross-table lateral radiographs to assess acetabular cup prominence.^5^^,^^15^ However, we have found that obtaining a false-profile view may be helpful to visualize an anteriorly protruded, vertical, or retroverted cup, and this can aid in visualizing overhanging heterotopic ossification that may impinge on the iliopsoas, as shown in Figure 3. To obtain a false-profile radiograph, the foot on the affected side is parallel to the x-ray plate and the pelvis is rotated 60° in relation to the leg. This view more appropriately allows the surgeon to evaluate anterior over-coverage, as well as acetabular cup protrusion and superior eccentric wear. In addition, magnetic resonance imaging may help diagnose pathology elsewhere, including fluid collections (abscesses), and can help determine implant stability. Furthermore, arthroscopy allows thorough assessment of the prosthetic components, and care should be taken to check the cup, liner, head ball, and neck at the Morse taper to assess for corrosion, wear, or loosening.

In summary, AII after THA is an uncommon problem that can be difficult to diagnose and treat. Obtaining a false-profile view is an effective method that can aid in diagnosis compared with the traditional cross-table lateral view. Conservative management using NSAIDs, physical therapy, and recurrent peritendinous injections of corticosteroids is the first line of treatment. If conservative management fails, arthroscopic iliopsoas lengthening is successful in providing safe and reliable resolution of symptoms, with considerable improvement in pain scores, as well as low complication rates. This surgery may also prevent the need for large acetabular revision operations.

### Limitations

We acknowledge the limitations of this study. We were limited in reporting only pain-score outcome measures because standardized patient-reported outcome measures are not routinely used at our institution. All of the cases included were performed by a single surgeon at a single academic institution with a small sample size without a control group, potentially limiting the generalizability of the results to the population at large. The retrospective design could introduce possible selection bias and data quality issues. There is relatively short-term follow-up, which could negate the ability to report complications that could occur at longer-term follow-up.

## Conclusions

Arthroscopic iliopsoas lengthening is a safe and effective treatment for AII after THA. The procedure had minimal complications, provided considerable pain relief in 80% of patients, and helped avoid major revision arthroplasty surgery in 100% of cases.

## Disclosures

All authors (J.P., A.H., D.M., T.M.G., A.W., B.C.) declare that they have no known competing financial interests or personal relationships that could have appeared to influence the work reported in this paper.
